# Evaluating verbal learning and memory in patients with an at-risk mental state or first episode psychosis using structural equation modelling

**DOI:** 10.1371/journal.pone.0196936

**Published:** 2018-05-10

**Authors:** Laura Egloff, Erich Studerus, Ronan Zimmermann, Ulrike Heitz, Stephanie Menghini-Müller, Sarah Ittig, Katharina Beck, Christina Andreou, Stefan Borgwardt, Anita Riecher-Rössler

**Affiliations:** 1 Department of Psychiatry, University of Basel Psychiatric Hospital, Basel, Switzerland; 2 Department of Psychology, Division of Clinical Psychology and Epidemiology, University of Basel, Basel, Switzerland; 3 Center for Gender Research and Early Detection, University of Basel Psychiatric Hospital, Basel, Switzerland; University of California Los Angeles, UNITED STATES

## Abstract

**Background:**

Verbal learning and memory are impaired not only in patients with a first episode of psychosis (FEP) but also–to a lower extent–in those with an at-risk mental state for psychosis (ARMS). However, little is known about the specific nature of these impairments. Hence, we aimed to study learning and memory processes in ARMS and FEP patients by making use of structural equation modelling.

**Methods:**

Verbal learning was assessed with the California Verbal Learning Test (CVLT) in 98 FEP patients, 126 ARMS patients and 68 healthy controls (HC) as part of the Basel early detection of psychosis (FePsy) study. The four-factorial CFA model of Donders was used to estimate test performance on latent variables of the CVLT and growth curve analysis was used to model the learning curve. The latter allows disentangling initial recall, which is strongly determined by attentional processes, from the learning rate.

**Results:**

The CFA model revealed that ARMS and FEP patients were impaired in *Attention Span*, *Learning Efficiency and Delayed Memory* and that FEP patients were additionally impaired in *Inaccurate Memory*. Additionally, ARMS-NT, but not ARMS-T, performed significantly worse than HC on *Learning Efficiency*. The growth curve model indicated that FEP patients were impaired in both initial recall and learning rate and that ARMS patients were only impaired in the learning rate.

**Conclusions:**

Since impairments were more pronounced in the learning rate than the initial recall, our results suggest that the lower scores in the CVLT reported in previous studies are more strongly driven by impairments in the rate of learning than by attentional processes.

## Introduction

The identification of so-called at-risk mental state for psychosis (ARMS) patients based on clinical signs [1, 2] is a promising approach for the early detection of psychotic disorders [3–5]. Neurocognitive impairments are a robust marker and considered to be core features of psychotic disorders, especially as these impairments persist even after remission of psychotic symptomatology [6, 7]. Cognitive impairments have been shown to be present in first episode psychosis (FEP) [8] as well as in ARMS patients [9–11] and may be useful for the prediction of transition to frank psychosis [3, 12].

Verbal learning and memory are among the most impaired cognitive functions in these patients and are therefore potentially useful as discriminatory variables in the early detection of psychotic disorders [4, 8, 13–15]. Previous studies on verbal learning and memory showed that FEP patients on average perform worse than ARMS and healthy controls (HC) and ARMS perform intermediate to FEP and HC [16]. Furthermore, ARMS patients who later transition to psychosis (ARMS-T) were shown to have poorer functioning in verbal memory in compared to ARMS without later transition to psychosis (ARMS-NT) [10, 11]. However, even though abundant literature is available on verbal learning and memory in chronic and FEP patients, only few studies with prospective design investigating ARMS patients have been published on verbal learning and memory so far [4, 5, 13, 17–20]. To the best of our knowledge, none of these studies focused on verbal learning and memory in more detail or did compare performances of FEP, ARMS patients with later transition to psychosis (ARMS-T) and HC directly in their analyses. Only four studies [20–23] so far investigated a sample of ARMS and FEP patients as well as HC in their analyses. However, none of these studies applied structural equation modelling.

Hence, the objective of this study was to evaluate group differences between ARMS, FEP and HC, as well as between ARMS-T and ARMS-NT, in regard to their performance on the California Verbal Learning Test (CVLT) [24] using the four factor structure as proposed by Donders [25] in a structural equation model. Furthermore, we aimed to investigate the learning curve of ARMS and FEP patients using latent growth curve modelling [26]. An important advantage of this approach is that it allows disentangling initial recall, which is strongly determined by attentional processes, from the rate of learning (i.e. learning slope) [27].

Based on the existing literature, we expected the sequence of performance on the CVLT to be the following: HC>ARMS>FEP and HC>ARMS-NT>ARMS-T>FEP, respectively.

## Materials and methods

### Setting and recruitment

The CVLT data analysed in this study were collected within the prospective ***F****rüh****e****rkennung von* ***Psy****chosen* (FePsy; early detection of psychosis) study, which aims to improve the early detection of psychosis. A more detailed description of the overall study design can be found elsewhere [3, 28]. Patients were recruited via the FePsy Clinic, University of Basel Psychiatric Hospital Basel, Switzerland, which was set up specifically to identify, assess, and treat individuals in the early stages of psychosis. All patients were recruited between March 2000 and November 2015. They were followed-up in regular intervals in order to distinguish those who later transitioned to psychosis (ARMS-T) from those who did not (ARMS-NT). During the first year of the follow-up ARMS individuals were assessed monthly for transition to psychosis, during the second and third year 3-monthly, and subsequently annually. HC were recruited from a commercial school, hospital staff and through advertisements and were not included if they had a current or former psychiatric disorder or neurological disease, serious medical condition, substance abuse, or a family history of psychiatric disorder. The study was approved by the ethics committee of North-western and Central Switzerland (EKNZ). All participants provided written informed consent.

### Screening procedure

The ARMS and FEP status was assessed using the Basel Screening Instrument for Psychosis (BSIP), which was developed by Riecher-Rössler, Aston [29]. The BSIP is based on the Personal Assessment and Crisis Evaluation (PACE) criteria by Yung, Philips [1] and has been shown to have a high predictive validity and a good inter reliability (κ = 0.67) [29]. Exclusion criteria were age younger than 18 years, insufficient knowledge of German, IQ <70, previous episode of psychosis (treated with antipsychotics for >3 weeks (lifetime) and a total amount of 2500mg chlorpromazine equivalent), psychosis clearly due to organic reasons or substance abuse, or psychotic symptoms within a clearly diagnosed affective psychosis or borderline personality disorder. Patients were identified as ARMS-NT if they had a follow-up period of at least three years without developing frank psychosis. ARMS-T’s time from first contact with the *FePsy* clinic until conversion to psychosis was 0.72 years in median (Mean = 1.18, SD = 1.32, Range = 0.03–4.64).

### Neurocognitive assessment

**Fig 1:**
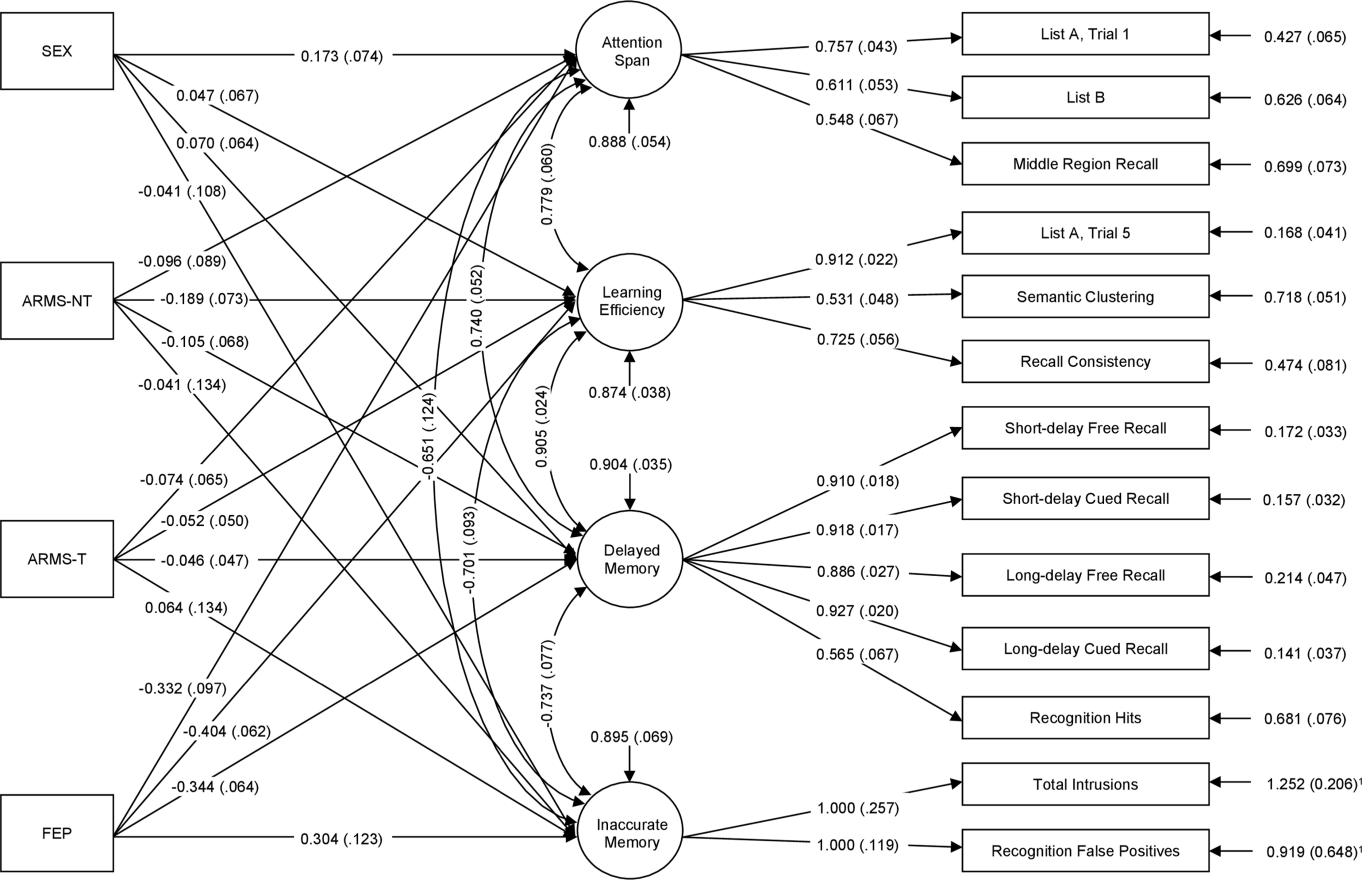
Visualisation of the confirmatory four factor analysis (CFA). The rectangles on the left represent the patient groups (at-risk mental state (ARMS) patients with (ARMS-T) and without later transition to psychosis (ARMS-NT) and first episode psychosis (FEP) patients). The circles in the middle represent the latent variables, which are measured by the indicator variables. The rectangles on the right represent the indicator variables, which consist out of observable data. Pathways are represented by arrows, showing the standardized XY estimates for each path. ^1^ Non standardized dispersion.

The CVLT is a widely used neurocognitive task which allows for a brief assessment of verbal learning strategies and processes. The test consists of two word lists each containing 16 words. List A is orally presented over five immediate-recall trials. An interference list (List B) is then presented for one immediate recall trial, followed by short- and long-delay free- and cued-recall and recognition test of List A. During the long-delay interval (approximately 20 min), nonverbal testing is administered to the subjects [24]. In confirmatory factor analyses (CFA) on the CVLT conducted by Donders [25] within a large standardisation sample with three different age groups of healthy controls as well as in a sample of traumatic brain injury patients [30] it has been demonstrated that a four factor structure containing the factors *Attention Span*, *Learning Efficiency*, *Delayed Memory*, and *Inaccurate Memory* provided good fit to the data. The variables measuring these factors are shown in Fig 1.

### Statistical analyses

All analyses were conducted using the R statistical environment, R version 3.2.3 [31] and Mplus Version 7 [32]. Sex was compared between groups with chi-square test. Age, years of education and BPRS total score were compared with independent t-test. Use of cannabis, antipsychotics, antidepressants, and anxiolytics were compared using Fisher’s Exact test. Cannabis use was not further included in the analyses since a former study by our research group found that there were no significant differences in neurocognitive performance between current, former, and never users, and there were no significant interactions between cannabis use and patient group [33].

Due to missing data in the outcome measures (see S1 Table), multiple imputations (MI) were performed using the Multivariate Imputation by Chained Equations software [34]. MI is considered the method of choice of handling complex incomplete data problems because it yields unbiased parameter estimates and standard errors under a missing at random (MAR) or missing completely at random (MCAR) missing data mechanism and maximizes statistical power by using all available information (Enders 2010). Although the MAR or MCAR assumption is not directly testable [35], it was considered plausible in the present situation because the variables with the highest proportion of missing values, such as the long delay free and cued recall, resulted from changes in the study design over the years and so the probability of being missing was unlikely to be directly dependent on the missing values themselves. Furthermore, even if the data were missing not at random, the MI procedure most likely would have led to less biased results than the traditional complete case analysis [36]. To protect against a potential power falloff from a too small number of imputations [37], we generated 20 imputations of the missing values using the random forest imputation method [38]. The analyses of interest (see below) were then conducted in each completed data set, and parameter estimates were pooled according to Rubin’s rules [39]. This method has previously been described by other members of this research group [33, 40].

In order to test CVLT performance differences between HC, ARMS-NT, ARMS-T and FEP patients directly within the structural equation modelling framework, we extended the measurement model of Donders [25] by regressing the four latent factors on group and sex. The variable group was represented in the model as three dummy coded contrast variables using the HC group as the reference group. This resulted in a so called multiple-indicator multiple-causes (MIMIC) model [41], which not only takes measurement error into account when testing group differences, but can also accommodate factorial non-invariance between group or so-called differential item functioning [41].

The following minimum standards have been specified a priori as desirable for the models: Comparative Fit Index (CFI) ≥0.95, Root Mean Squared Error of Approximation (RMSEA) ≤0.06, Tucker-Lewis Index (TLI) ≥0.95, Standardized Root Mean Square Residual (SRMR) ≤0.08 [42].

**Fig 2:**
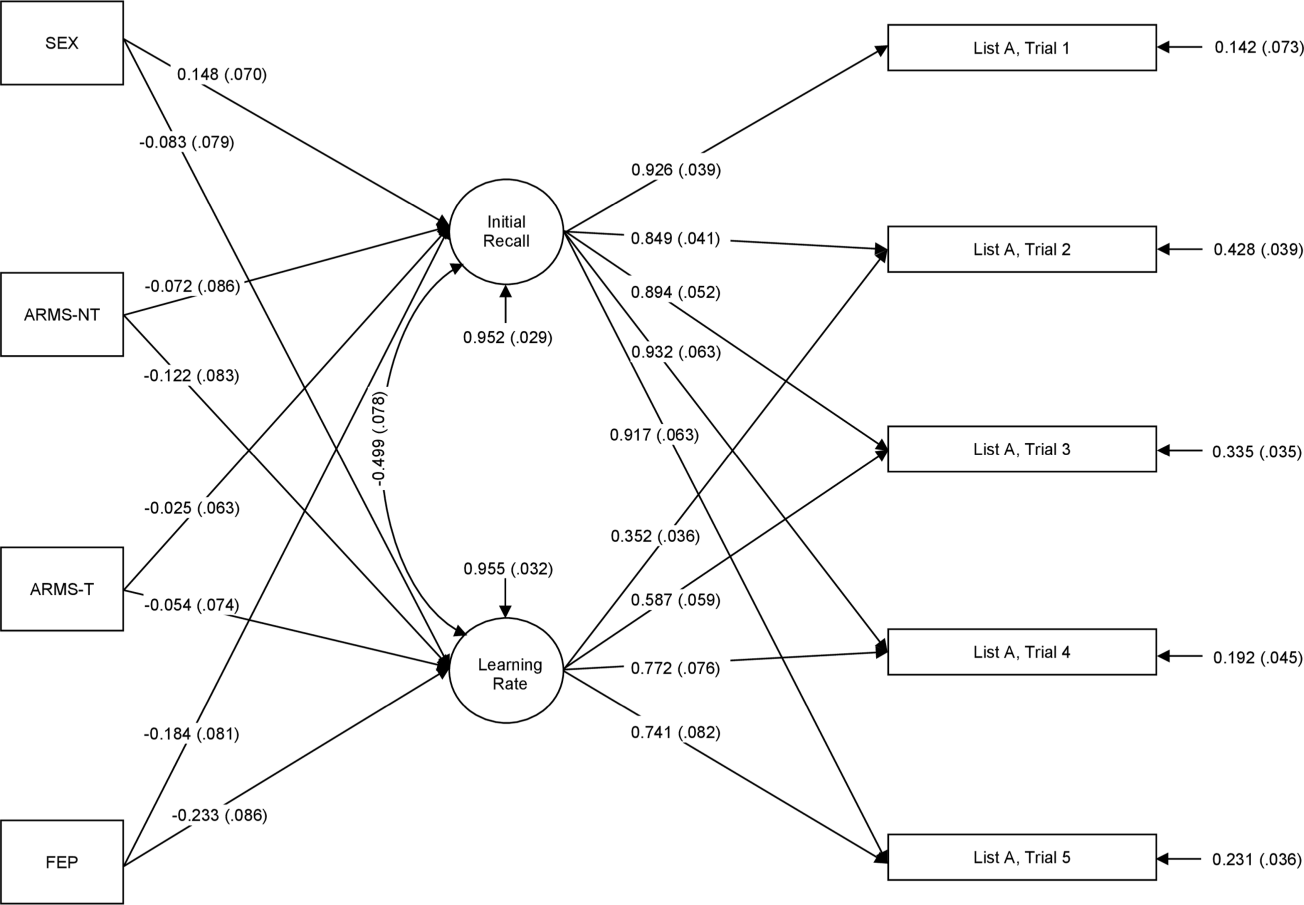
Visualisation of the growth curve analysis. The rectangles on the left represent the patient groups (at-risk mental state (ARMS) patients with (ARMS-T) and without later transition to psychosis (ARMS-NT) and first episode psychosis (FEP) patients). The circles in the middle represent the latent variables, which are measured by the indicator variables. The rectangles on the right represent the indicator variables, which consist out of observable data. Pathways are represented by arrows, showing the standardized XY estimates for each path.

Second, learning over the first 5 trials of the CVLT was investigated using latent growth curve analysis which is a statistical technique used in the SEM framework to estimate growth trajectories. Several studies have successfully applied growth curve models on the CVLT or the closely related Rey Auditory Verbal Learning Task (RAVLT) [43–45]. An important advantage of this approach is that it allows disentangling initial recall, which is strongly determined by attentional processes, from the rate of learning (i.e., learning slope). Thus, we used the two latent parameters *initial recall* and *learning rate* as proposed by Jones, Rosenberg [45] to model the growth curve. Here, *initial recall* corresponds to the intercept and *learning rate* corresponds to the slope of the growth curve. Both parameters were estimated based on the number of recalled words over the first five trials of the CVLT (see Fig 2).

All analyses were first conducted with the three groups ARMS, FEP and HC. Thereafter, analyses were extended to the subgroups ARMS-T and ARMS-NT, compared to FEP and HC respectively. In a further step, we conducted secondary analyses for the MIMIC model proceeding exactly as described above but including the covariates that differed significantly between groups for completeness.

To account for multiple testing corrected *p* values were calculated using the false discovery rate [46].

## Results

98 FEP patients, 126 ARMS and 68 HC fulfilled the inclusion criteria (see Table 1 for sociodemographic and clinical sample characteristics). Of the 126 ARMS patients, 25 had developed frank psychosis during the follow-up (ARMS-T), 48 had not developed psychosis after a follow up of at least 3 years (ARMS-NT), and 53 had not developed psychosis but were followed up for less than 3 years either because they were only recruited recently or because they dropped out during the first three years of the follow-up.

**Table 1:** Sociodemographic and clinical sample characteristics.

	N	HC	ARMS			FEP	Test Statistic HC vs. ARMS vs. FEP
		*N* = 68	*N* = 126			*N* = 98	
				ARMS-NT	ARMS-T		
				*N* = 48	*N* = 25		
Gender	292						*p* = 0.226^1^
*Female*		27 (40%)	36 (28.6%)	13 (27.1%)	10 (40%)	36 (36.7%)	
*Male*		41 (60%)	90 (71.4%)	35 (72.9%)	15 (60%)	62 (63.3%)	
Age	292	25.30 ± 6.04	25.6 ± 6.56	25.7 ± 7.32	26.85 ± 6.97	28.5 ± 8.16	*p* = 0.003^2^
Years of education	291	13.19 ± 2.84	11.8 ± 2.81	11.9 ± 3.29	11.40 ± 2.02	11.5 ± 2.92	*p* = 0.001^2^
Antipsychotics currently:	292	0 (0%)	9 (7.14%)	0 (0%)	3 (12%)	36 (36.7%)	*p* < 0.001^3^
Antidepressants currently:	292	0 (0%)	43 (34.1%)	20 (41.7%)	9 (36%)	18 (18.4%)	*p* < 0.001^3^
Anxiolytics currently:	292	0 (0%)	19 (15.1%)	5 (10.4%)	7 (28%)	19 (19.4%)	*p* = 0.001^3^
Cannabis use:	244	5 (8%)	24 (23.5%)	9 (28%)	4 (22%)	22 (26.5%)	*p* = 0.023^3^
BPRS total score	205		38.6 ± 9.13	37.3 ± 9.01	41.39 ± 8.82	50.8 ± 12.5	*p* < 0.001^2^

*Note*. N is the number of non-missing values. Values of continuous variables are stated as mean ± 1 standard deviation.

^1^Pearson’s χ^2^ test

^2^independent t-test

^3^Fisher’s Exact test.

HC = healthy controls; ARMS = patients with an at-risk mental state for psychosis; ARMS-NT = patients with an at-risk mental state for psychosis without later transition to psychosis; ARMS-T = patients with an at-risk mental state for psychosis with later transition to psychosis; FEP = patients with a first episode of psychosis. Patients were identified as ARMS-NT if they had a follow-up period of at least three years without developing frank psychosis.

### Confirmatory factor analysis (CFA)

The four factorial CFA model of Donders [25] narrowly missed acceptability of model fit in our data (χ^*2*^ = 180.988, df = 59, *p* < 0.001, *AIC* = 10852.479, *CFI* = 0.921, RMSEA = 0.093, TLI = 0.895, SRMR = 0.045). In addition, an error warning indicated that the covariance matrix of latent variables was not positive definite.

We therefore made a small modification of the model of Donders by treating the indicators of the Inaccurate Recall factor (i.e. total intrusions and recognition false positives) as count variables and estimating their loadings on the Inaccurate Recall factor through zero-inflated negative binomial regressions. This not only solved the convergence problem, it also led to a more realistic data model as total intrusions and recognition false positives were now correctly modelled as non-negative integers with relatively large preponderances of zeros. Unfortunately, as this new model could only be estimated by numerical integration [47], traditional fit indices were not available for this modified model. However, the Akaike Information Criterion (AIC) indicated the modification led to an improved model fit (*AIC*_*OLD*_ = 10852.479, *AIC*_*NEW*_ = 10050.095).

Results from the MIMIC model showed significant worse performance of ARMS and FEP on *Attention Span*, *Learning Efficiency* and *Delayed Memory* compared to HC. Additionally, FEP showed significantly worse performance on *Inaccurate Memory* than HC (see Table 2).

**Table 2:** Results from the four factor multiple-indicator multiple-causes (MIMIC) model.

		FEP		ARMS		ARMS-NT		ARMS-T		SEX	
		Estimate (S.E.)	*p*-value	Estimate (S.E.)	*p*-value	Estimate (S.E.)	*p*-value	Estimate (S.E.)	*p*-value	Estimate (S.E.)	*p*-value
Comparison of FEP and ARMS vs. HC											
	Attention Span	-0.33 (0.09)	<0.001***	-0.16 (0.08)	0.048*					0.25 (0.07)	<0.001***
	Learning Efficiency	-0.39 (0.06)	<0.001***	-0.22 (0.06)	<0.001***					0.09 (0.06)	0.135
	Delayed Memory	-0.32 (0.06)	<0.001***	-0.13 (0.06)	0.028*					0.11 (0.06)	0.041*
	Inaccurate Memory	0.33 (0.12)	0.006**	0.02 (0.12)	0.870					-0.03 (0.10)	0.797
Comparison of FEP, ARMS-NT and ARMS-T vs. HC											
	Attention Span	-0.33 (0.10)	0.001***			-0.10 (0.09)	0.284	-0.07 (0.07)	0.256	0.17 (0.07)	0.020*
	Learning Efficiency	-0.40 (0.06)	<0.001***			-0.19 (0.07)	0.009**	-0.05 (0.05)	0.302	0.05 (0.07)	0.481
	Delayed Memory	-0.34 (0.06)	<0.001***			-0.11 (0.07)	0.123	-0.05 (0.05)	0.332	0.07 (0.06)	0.274
	Inaccurate Memory	0.30 (0.12)	0.014*			-0.04 (0.13)	0.762	0.06 (0.13)	0.634	-0.04 (0.11)	0.708
Comparison of ARMS-NT vs. ARMS-T											
	Attention Span					0.00 (0.10)	0.971			0.17 (0.07)	0.021*
	Learning Efficiency					-0.12 (0.09)	0.165			0.05 (0.07)	0.486
	Delayed Memory					-0.04 (0.08)	0.581			0.07 (0.06)	0.279
	Inaccurate Memory					-0.14 (0.19)	0.474			-0.04 (0.11)	0.731

*Note*. S.E. = Standard Error.

Level of significance for p-values

*p ≤ 0.05

**p ≤ 0.01

***p ≤ 0.001.

ARMS = patients with an at-risk mental state for psychosis; ARMS-NT = patients with an at-risk mental state for psychosis without later transition to psychosis; ARMS-T = patients with an at-risk mental state for psychosis with later transition to psychosis; FEP = patients with a first episode of psychosis. Patients were identified as ARMS-NT if they had a follow-up period of at least three years without developing frank psychosis. To account for multiple testing corrected *p*-values were calculated using the false discovery rate.

**Fig 3:**
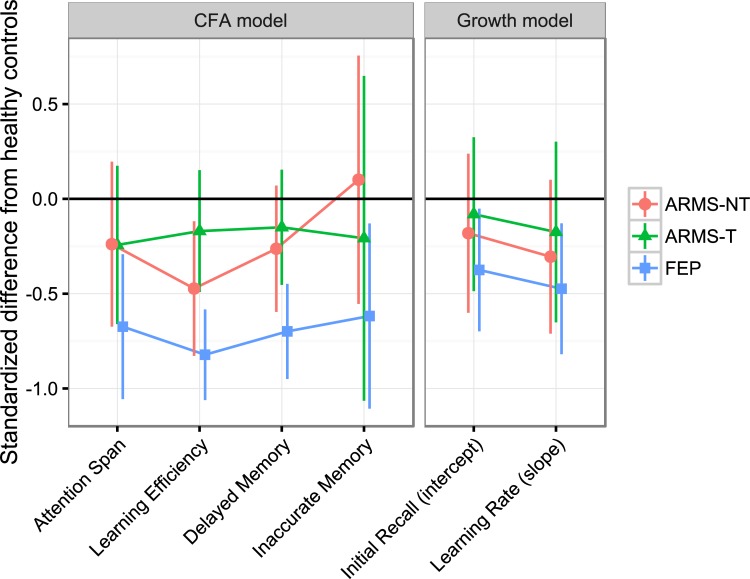
Performances of at-risk mental state (ARMS) patients with (ARMS-T) and without later transition to psychosis (ARMS-NT) and first episode psychosis (FEP) patients on the four factors of the California Verbal Learning Test (CVLT). The horizontal line at zero represents the performance of healthy controls. Differences are expressed in units of standardized mean differences. Differences are significant if the 95% confidence interval does not overlap with the horizontal line. The variable *Inaccurate Memory* was reversed such that high scores represent a good performance. Differences are adjusted for the influence of sex.

When splitting up the ARMS group into ARMS-T and ARMS-NT only ARMS-NT showed a significant worse performance than HC on *Learning Efficiency* (*p* = 0.009). FEP still showed significant worse performance on all four factors compared to HC (*p*_*Attention Span*_ = 0.001, *p*_*Learning Efficiency*_ < 0.001, *p*_*Delayed Memory*_ < 0.001, *p*_*Inaccurate Memory*_ = 0.014) (see Table 2 and Fig 3). When comparing ARMS-T against ARMS-NT, no significant differences emerged on any of the four factors (see Table 2).

Results from the secondary MIMIC model analyses showed no significant differences in the performances of ARMS, ARMS-NT, ARMS-T and FEP on any of the investigated latent variables compared to HC. The only covariates significantly influencing the latent factors were sex and years of education, indicating that women perform significantly better than men on *Attention Span* and those subjects with more years of education exhibiting superior performances on *Learning Efficiency* and *Delayed Memory*, independent of diagnostic group (see Tables 3–5). A stepwise integration of the covariates revealed that the performance differences between ARMS patients and HC were no longer significant when corrected for years of education and the performance differences between FEP patients and HC were no longer significant when corrected for use of antipsychotics.

**Table 3:** Results from the secondary four-factor multiple-indicator multiple-causes (MIMIC) model including covariates.

		FEP		ARMS		ARMS-NT		ARMS-T		SEX	
		Estimate (S.E.)	*p*-value	Estimate (S.E.)	*p*-value	Estimate (S.E.)	*p*-value	Estimate (S.E.)	*p*-value	Estimate (S.E.)	*p*-value
Comparison of FEP and ARMS vs. HC											
	Attention Span	-0.206 (0.104)	0.188	-0.110 (0.092)	0.496					0.258 (0.066)	<0.001***
	Learning Efficiency	-0.173 (0.083)	0.167	-0.153 (0.068)	0.162					0.082 (0.057)	0.378
	Delayed Memory	-0.095 (0.088)	0.506	-0.014 (0.068)	0.857					0.113 (0.054)	0.167
	Inaccurate Memory	0.148 (0.158)	0.524	-0.141 (0.135)	0.506					-0.025 (0.102)	0.857
Comparison of FEP, ARMS-NT and ARMS-T vs. HC											
	Attention Span	-0.180 (0.112)	0.393			-0.049 (0.094)	0.797	0.008 (0.075)	0.965	0.180 (0.073)	0.187
	Learning Efficiency	-0.162 (0.089)	0.393			-0.132 (0.073)	0.393	0.037 (0.058)	0.719	0.044 (0.063)	0.717
	Delayed Memory	-0.132 (0.091)	0.403			-0.028 (0.073)	0.832	0.059 (0.059)	0.578	0.073 (0.062)	0.510
	Inaccurate Memory	0.130 (0.168)	0.714			-0.153 (0.152)	0.578	-0.050 (0.143)	0.832	-0.037 (0.110)	0.832
Comparison of ARMS-NT vs. ARMS-T											
	Attention Span					-0.061 (0.098)	0.712			0.180 (0.073)	0.149
	Learning Efficiency					-0.182 (0.083)	0.186			0.045 (0.063)	0.698
	Delayed Memory					-0.106 (0.079)	0.443			0.073 (0.062)	0.454
	Inaccurate Memory					-0.082 (0.199)	0.827			-0.037 (0.111)	0.827

*Note*. S.E. = Standard Error.

Level of significance for p-values

*p ≤ 0.05

**p ≤ 0.01

***p ≤ 0.001.

ARMS = patients with an at-risk mental state for psychosis; ARMS-NT = patients with an at-risk mental state for psychosis without later transition to psychosis; ARMS-T = patients with an at-risk mental state for psychosis with later transition to psychosis; FEP = patients with a first episode of psychosis. Patients were identified as ARMS-NT if they had a follow-up period of at least three years without developing frank psychosis. To account for multiple testing corrected *p*-values were calculated using the false discovery rate.

**Table 4:** Results from the secondary four-factor multiple-indicator multiple-causes (MIMIC) model including covariates.

		Years of Education		BPRS Total Score		Cannabis	
		Estimate (S.E.)	*p*-value	Estimate (S.E.)	*p*-value	Estimate (S.E.)	*p*-value
Comparison of FEP and ARMS vs. HC							
	Attention Span	0.167 (0.061)	0.054	-0.059 (0.096)	0.640	0.046 (0.077)	0.640
	Learning Efficiency	0.199 (0.055)	<0.001***	-0.130 (0.083)	0.357	-0.062 (0.066)	0.524
	Delayed Memory	0.157 (0.051)	0.024*	-0.136 (0.079)	0.275	-0.017 (0.061)	0.857
	Inaccurate Memory	-0.136 (0.106)	0.450	0.116 (0.116)	0.517	0.089 (0.098)	0.524
Comparison of FEP, ARMS-NT and ARMS-T vs. HC							
	Attention Span	0.136 (0.068)	0.368	-0.130 (0.100)	0.485	0.038 (0.089)	0.832
	Learning Efficiency	0.215 (0.061)	<0.001***	-0.156 (0.096)	0.393	-0.098 (0.080)	0.498
	Delayed Memory	0.172 (0.055)	0.040*	-0.132 (0.090)	0.403	-0.023 (0.073)	0.832
	Inaccurate Memory	-0.089 (0.125)	0.717	0.202 (0.125)	0.393	0.038 (0.116)	0.832
Comparison of ARMS-NT vs. ARMS-T							
	Attention Span	0.136 (0.068)	0.240	-0.130 (0.100)	0.443	0.038 (0.089)	0.827
	Learning Efficiency	0.216 (0.061)	<0.001***	-0.155 (0.096)	0.384	-0.098 (0.080)	0.448
	Delayed Memory	0.173 (0.055)	0.032*	-0.132 (0.090)	0.387	-0.023 (0.073)	0.827
	Inaccurate Memory	-0.092 (0.126)	0.698	0.201 (0.125)	0.384	0.038 (0.116)	0.827

*Note*. S.E. = Standard Error.

Level of significance for p-values

*p ≤ 0.05

**p ≤ 0.01

***p ≤ 0.001.

ARMS = patients with an at-risk mental state for psychosis; ARMS-NT = patients with an at-risk mental state for psychosis without later transition to psychosis; ARMS-T = patients with an at-risk mental state for psychosis with later transition to psychosis; FEP = patients with a first episode of psychosis. Patients were identified as ARMS-NT if they had a follow-up period of at least three years without developing frank psychosis. Cannabis refers to current use. To account for multiple testing corrected *p*-values were calculated using the false discovery rate.

**Table 5:** Results from the secondary four-factor multiple-indicator multiple-causes (MIMIC) model including covariates.

		Antipsychotics		Antidepressants		Anxiolytics	
		Estimate (S.E.)	*p*-value	Estimate (S.E.)	*p*-value	Estimate (S.E.)	*p*-value
Comparison of FEP and ARMS vs. HC							
	Attention Span	-0.048 (0.073)	0.640	0.050 (0.072)	0.640	-0.101 (0.072)	0.393
	Learning Efficiency	-0.170 (0.077)	0.162	0.064 (0.058)	0.506	0.006 (0.067)	0.931
	Delayed Memory	-0.136 (0.076)	0.260	-0.040 (0.062)	0.640	-0.076 (0.067)	0.506
	Inaccurate Memory	-0.024 (0.111)	0.857	0.149 (0.101)	0.378	0.057 (0.096)	0.640
Comparison of FEP, ARMS-NT and ARMS-T vs. HC							
	Attention Span	0.002 (0.088)	0.983	-0.005 (0.082)	0.978	-0.167 (0.075)	0.260
	Learning Efficiency	-0.138 (0.090)	0.403	0.018 (0.066)	0.852	-0.059 (0.078)	0.714
	Delayed Memory	-0.083 (0.083)	0.578	-0.055 (0.069)	0.714	-0.126 (0.073)	0.393
	Inaccurate Memory	-0.081 (0.121)	0.719	0.173 (0.121)	0.403	0.127 (0.102)	0.498
Comparison of ARMS-NT vs. ARMS-T							
	Attention Span	0.002 (0.088)	0.984	-0.005 (0.082)	0.982	-0.167 (0.075)	0.186
	Learning Efficiency	-0.138 (0.090)	0.387	0.017 (0.066)	0.845	-0.060 (0.078)	0.698
	Delayed Memory	-0.083 (0.083)	0.562	-0.055 (0.069)	0.698	-0.126 (0.073)	0.375
	Inaccurate Memory	-0.081 (0.121)	0.698	0.176 (0.121)	0.387	0.128 (0.103)	0.448

*Note*. S.E. = Standard Error. ARMS = patients with an at-risk mental state for psychosis; ARMS-NT = patients with an at-risk mental state for psychosis without later transition to psychosis; ARMS-T = patients with an at-risk mental state for psychosis with later transition to psychosis; FEP = patients with a first episode of psychosis. Patients were identified as ARMS-NT if they had a follow-up period of at least three years without developing frank psychosis. Antipsychotics, Antidepressants, and Anxiolytics refer to current use. To account for multiple testing corrected *p*-values were calculated using the false discovery rate.

### Growth curve analysis

**Fig 4:**
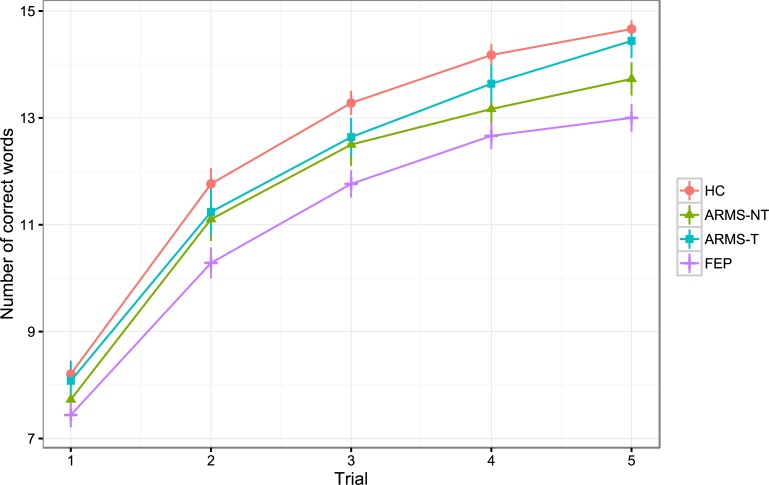
Growth curve. Verbal learning performances of at-risk mental state (ARMS) patients with (ARMS-T) and without later transition to psychosis (ARMS-NT) and first episode psychosis (FEP) patients. Lines per group correspond to the mean of total words remembered per trial.

A comparison of three nested models with different shapes for the learning curve revealed that an approximately logarithmic growth curve (χ^*2*^ = 51.361, df = 15, *p* < 0.001, *AIC* = 6414.138, *CFI* = 0.960, RMSEA = 0.089, TLI = 0.939, SRMR = 0.061) and a freely estimated growth curve (χ^*2*^ = 34.258, df = 13, *p* = 0.001, *AIC* = 6399.236, *CFI* = 0.977, RMSEA = 0.074, TLI = 0.964, SRMR = 0.055) provided both good fit to the data. Hence, for ease of interpretation, the approximately logarithmic model was used for comparing *initial recall* and *learning* rate of ARMS and FEP patients. FEP showed significantly lower scores in *initial recall* (*p* = 0.021) and *learning rate* (*p* = 0.010) compared to ARMS and HC (see Fig 4). Additionally, a significantly worse performance of ARMS compared to HC was found regarding *learning rate* (*p* = 0.050). To further distinguish between ARMS-T and ARMS-NT a second growth curve analysis with these two subgroups was conducted. Results showed significant worse performances of FEP compared to HC in *initial recall* (*p* = 0.024) and *learning rate* (*p* = 0.007). No significant differences were found for ARMS-T and ARMS-NT compared to HC on these two factors (ARMS-T: *p*_*Initial Recall*_ = 0.695, *p*_*Learning Rate*_ = 0.471; ARMS-NT: *p*_*Initial Recall*_ = 0.398, *p*_*Learning Rate*_ = 0.142).

Also, when comparing ARMS-T against ARMS-NT no significant group differences emerged (*p*_*Initial Recall*_ = 0.433, *p*_*Learning Rate*_ = 0.193).

## Discussion

This study examined learning curves in at-risk mental state (ARMS) patients, first episode psychosis (FEP) patients and healthy controls (HC) using structural equation modelling. We hypothesized that HC would perform best on all four factors of the CVLT, ARMS would perform intermediate to HC and FEP and that FEP would perform worst compared to HC and ARMS (i.e. HC>ARMS>FEP). This hypothesized sequence of performance was confirmed within the MIMIC model for the three out of four factors *Attention Span*, *Learning Efficiency* and *Delayed Memory* of the CVLT. Additionally, growth curve analysis verified this sequence of performance for the latent factor *Learning Rate*.

When further differentiating the ARMS patient group into ARMS with later transition to psychosis (ARMS-T) and ARMS without later transition to psychosis (ARMS-NT), only ARMS-NT showed a significantly worse performance on the factor *Learning Efficiency* compared to HC. No further group differences between ARMS-T and ARMS-NT were found, neither within the MIMIC model nor in the growth curve analysis.

The herein found results show that impairments in verbal learning and memory in FEP patients are present in the domains of *Attention Span*, *Learning Efficiency*, *Delayed Memory* and *Inaccurate Memory*. Likewise, ARMS patients show similar impairments as FEP patients although less marked in the domains *Attention Span*, *Learning Efficiency* and *Delayed Memory*. Furthermore, it was shown that regarding the verbal learning curve only FEP patients demonstrated marked impairments in the initial recall (i.e. intercept of the slope), whereas in the learning rate both FEP and ARMS patients showed significant impairments compared to HC. Nevertheless, when representing the verbal learning curve graphically it became apparent from the development of the curve per group over the five trials of the CVLT, that the learning curve progresses similarly for each group (see Fig 4). More precisely, the development of the growth curve, given the increase of the slope, indicates that ARMS and FEP perform similarly to HC in regard to the Learning Rate, even though the increase of the curve is less steep than the one of the HC. It has to be emphasized that, in contrast to most previous studies, this study does not rely solely on Trial 1, Trial 5 or the cumulative sum (i.e. sum of trials 1–5) of the CVLT but evaluates each of the five trials on its own, taking all available information into account.

The results from the confirmatory factor analysis (CFA) are in line with the present literature indicating impairments of attentional processing in ARMS and FEP, with ARMS performing intermediate to HC and FEP, and FEP performing worse than the other groups [16, 48].

Furthermore, the results correspond to the findings of the meta-analysis conducted by Bora, Lin [11] who reported medium effect sizes for deficits of ARMS patients in verbal memory (*d* = -.50) and in verbal learning (*d* = -.68). Giuliano, Li [49], who found similar effect sizes, suggested that it may be possible to improve the predicted trajectory of psychotic illnesses by integrating information about specific cognitive deficit patterns such as in the verbal declarative memory. Accordantly, Fusar-Poli, Deste [10] based on their meta-analysis suggest that ARMS patients are impaired in tests of verbal memory and later transition to psychosis is associated with poorer verbal memory. Congruently, Koutsouleris, Davatzikos [4] found that transition to psychosis was mainly predicted by executive and verbal learning impairments.

In contrast to our hypothesis, ARMS-NT, but not ARMS-T, performed significantly worse on the factor *Learning Efficiency* compared to HC. This rather counterintuitive result may on the one hand be due to the fact that the ARMS-T sample was too small to detect significant differences between groups (N_ARMS-T_ = 25, N_ARMS-NT_ = 48). On the other hand, a plausible explanation might be that impaired *Learning Efficiency* represents a feature/trait of the at-risk mental state but is not related to the onset of illness.

On all other factors no significant differences between ARMS-T and ARMS-NT emerged, therefore it may be speculated that only the overall performance in the domain of verbal memory may be predictive of a later transition to frank psychosis, but that single underlying factors of verbal learning and memory are not. This assumption would be in line with the results of Francey, Jackson [50] who showed that, despite the existence of sustained attention deficits in ARMS, there were no differences between subjects who converted to psychosis (ARMS-T) and those who did not (ARMS-NT). This led the authors to suggest that such deficits, although they may constitute a vulnerability factor, are insufficient for reliable predictions of the risk of conversion to psychosis. Also, a meta-analysis by De Herdt, Wampers [51] indicated that ARMS-T and ARMS-NT may not be differentiated based on verbal memory deficits. Furthermore, in their meta-analysis Bora, Lin [11] reported that effect sizes for between-group differences were modest with a Cohen’s *d* = 0.5 at most for domains with the largest group differences (i.e. verbal fluency, verbal and visual memory, and working memory). Thus, the authors found a significant performance overlap of 67% between the groups, indicating that cognitive impairment has only a limited capacity to predict the outcome of high-risk patients.

However, when the analyses were repeated including all covariates significantly differing between groups, no significant group differences regarding performance on any of the latent variables could be observed anymore. A stepwise integration revealed that this was specifically due to the covariates years of education and antipsychotic medication. However, it could be argued that it is not sensible to correct for the influence of years of education here, because reduced years of education can be consequence of the psychotic disorder [52] and thus one would partial out variability of the illness itself. The same is true for antipsychotic medication, which was not present in HC, but in 7.1% of ARMS and 36.7% of FEP patients.

In line with our hypothesis, the results from the growth curve analysis indicated a worse performance of FEP compared to ARMS and HC and a performance of ARMS intermediate to those two groups. Since these differences were more pronounced in the slope (i.e. learning rate) than in the intercept (i.e. initial recall) of the learning curve, our results are in line with the existing body of literature indicating that the verbal learning rate tends to be more impaired than attentional processes in both ARMS and FEP patients [10, 11, 14].

Additionally, the results suggest that the worse performance in verbal learning and memory in FEP patients is due to both a lower initial recall and a lower learning rate pointing towards a possible underlying attentional problem in FEP. ARMS patients showed a similar pattern of impairment as FEP regarding learning rate. This finding is consistent with recently published reviews and meta-analyses indicating that already in the prodromal/at-risk mental state patients show marked impairments in verbal memory [10, 11, 48].

### Limitations

The following limitations should be taken into account:

Firstly, sample sizes differed across groups. Particularly, when differentiating between ARMS-T and ARMS-NT moderate group sizes emerged. Literature suggests cognitive impairments in ARMS-T patients to be rather unspecific and generalized [12]. Hence, the small and distinct group sizes may have precluded the detection of small effects between these two groups. Furthermore, the probability of a type II error is considerably larger for comparisons of ARMS-T and ARMS-NT than for comparisons between ARMS, FEP and HC groups.

Secondly, to be identified as non-transitioned patients had to be in the follow-up for at least three years without transitioning to psychosis. Although research has shown that most ARMS-T patients make the transition to psychosis within the first 12 months of clinical presentation, a small percentage of patients transitions to frank psychosis within the next 24 months of follow-up (for meta-analysis, see [10]). This cut-off contributed substantially to the small sample size of the ARMS-NT group. Yet, by setting this cut-off we were able to strongly decrease the risk of misclassifying patients with a later transition to psychosis as non-transitioned cases.

Thirdly, the measurement model of Donders [25] used in our analyses was originally built based on the standardization sample data of the CVLT-II, whereas in this study we had measured verbal learning and memory with the original CVLT. However, we could still fit the model of Donders with our CVLT data because all required indicator variables are also available in the original CVLT. The only difference is that Semantic Clustering is calculated slightly differently. However, since we calculated Semantic Clustering according to the CVLT-II instructions, we do not expect that the application of a CVLT-II model to our CVLT data has largely influenced the results.

Fourthly, since HC were not included if they had a current or former psychiatric disorder or neurological disease, we cannot rule out the possibility that our HC were healthier than a representative sample from the general population and that consequently differences between HC and patients were overestimated.

### Conclusion

In conclusion, this is the first study to evaluate verbal learning and memory using structural equation modelling. In line with our hypothesis, results indicated a worse performance of FEP patients compared to ARMS patients and HC and a performance of ARMS patients intermediate to those two groups. Since these differences were more pronounced in the slope than in the intercept of the learning curve, our results indicate that the verbal learning rate tends to be more impaired than attentional processes in both ARMS and FEP patients. Further longitudinal investigations are needed to clarify whether verbal learning and memory may be a potential discriminatory variable in the early detection of psychosis.

## Supporting information

S1 Table Summary of missing values in each variable. *Note*. CVLT = California Verbal Learning Task. Missing Values resulted from changes in the study design over the years.
(DOCX)
