# Supplementary material for: Evaluating verbal learning and memory in patients with an at-risk mental state or first episode psychosis using structural equation modelling
Source: PLoS One. 2018 May 10;13(5):e0196936. doi: 10.1371/journal.pone.0196936 (PMC5944996; doi:10.1371/journal.pone.0196936)
Supplement: S1 Table — Note. CVLT = California Verbal Learning Task. Missing Values resulted from changes in the study design over the years. (DOCX) [file pone.0196936.s001.docx]

**S1Table.** Summary of missing values in each variable

|  | N missings | % missings |
| --- | --- | --- |
| **Verbal Learning & Memory** | | |
| CVLT List A, Trial 1 | 0 | 0 |
| CVLT List B | 1 | 0.34 |
| CVLT Middle Region Recall | 0 | 0 |
| CVLT List A, Trial 5 | 0 | 0 |
| CVLT Semantic Clustering | 0 | 0 |
| CVLT Recall Consistency | 0 | 0 |
| CVLT Short-delay Free Recall | 1 | 0.34 |
| CVLT Short-delay Cued Recall | 1 | 0.34 |
| CVLT Long-delay Free Recall | 45 | 15.41 |
| CVLT Long-delay Cued Recall | 45 | 15.41 |
| CVLT Recognition Hits | 46 | 15.75 |
| CVLT Total Intrusions | 46 | 15.75 |
| CVLT Recognition False Positives | 46 | 15.75 |

*Note.* CVLT = California Verbal Learning Task. Missing Values resulted from changes in the study design over the years.
